# The genetic relationship between hypotension and delirium: a Mendelian randomization study

**DOI:** 10.3389/fneur.2024.1408956

**Published:** 2024-07-17

**Authors:** Chengli Wang, Jiayao Wu, Yiqing Lin, Zhongqi Liu, Ning Liufu, Minghui Cao

**Affiliations:** ^1^Department of Anesthesiology, Shenshan Medical Center, Sun Yat-sen Memorial Hospital, Sun Yat-sen University, Shanwei, Guangdong, China; ^2^Guangdong Provincial Key Laboratory of Malignant Tumor Epigenetics and Gene, Regulation, Sun Yat-sen Memorial Hospital, Sun Yat-sen University, Guangzhou, China; ^3^Department of Anesthesiology, Guangdong Women and Children Hospital, Guangzhou, China

**Keywords:** Mendelian randomization, hypotension, delirium, genetic, causal association

## Abstract

**Background:**

Observational research suggests that hypotension is a potential hazard factor of delirium. Nevertheless, previous observational articles are limited in their ability to establish causality between hypotension and delirium. The present study was sought to explore the genetic causal relationship between these two conditions using two-sample Mendelian randomization (MR).

**Methods:**

Genome-wide association study (GWAS) summarized data for hypotension and delirium were obtained from the FinnGen Consortium. The researchers utilized several statistical methods, such as inverse-variance weighted (IVW), weighted median, MR Egger, weighted mode, and simple mode in conducting the MR statistical analysis. In order to identify heterogeneity among the MR outcomes, we employed the Cochrane’s Q test. Furthermore, we used the MR-Egger intercept test and MR pleiotropy residual sum and outliers (MR-PRESSO) test to examine horizontal pleiotropy.

**Results:**

The findings revealed that hypotension was identified as an independent hazard variable for delirium (*p* = 0.010, odds ratio [OR] [95% confidence interval (CI)] = 1.302 [1.066–1.592]) using the IVW method. The presence of horizontal pleiotropy was found to have minimal impact on establishing causal relationship (*p* = 0.999), and there was no evidence to suggest heterogeneity between genetic variations (*p* = 0.379). Additionally, the leave-one-out method demonstrated the stability and robustness of this association.

**Conclusion:**

We performed two-sample MR analyses and found evidence of a genetic causal relationship between hypotension and delirium. Our findings suggest that individuals with a genetic predisposition for hypotension may have a higher risk of developing delirium. This suggests that interventions aimed at improving perioperative hypotension could aid in limiting the incidence of delirium.

## 1 Introduction

Delirium, characterized by a sudden decline and fluctuation in attention, cognitive function, and consciousness, is the prevailing psychiatric syndrome encountered in older adults admitted to hospitals following acute illness or surgery (1). Its occurrence varies between 11% and 51%, contingent upon the specific setting or patient demographic (2, 3). Delirium is linked to protracted hospital stays, increased morbidity and mortality rates, cognitive deterioration, the onset of dementia, and inferior overall outcomes. Given the absence of efficacious interventions, it is imperative to identify modifiable risk factors and implement preventive measures (4–6).

During surgical procedures, hypotension commonly manifests, leading to a potential reduction in the supply of oxygen and blood to essential organs, resulting in injuries to the heart, kidneys, and brain (7). It has been proposed that intraoperative hypotension could diminish cerebral perfusion, thereby disrupting brain homeostasis and potentially causing brain injury, ultimately leading to delirium (8–10). The brain exhibits a distinct reserve capacity in its response to challenges of ischemia and hypoxia in both young individuals and adults. However, this capacity is diminished in elderly individuals under general anesthesia, primarily due to their compromised arterial elasticity and presence of comorbidities (11). It has been observed that a specific duration of hypotension during surgical procedures can lead to brain damage and subsequent neurological complications, such as delirium (12, 13). Nevertheless, there exists a contentious debate regarding the association between hypotension and the occurrence of delirium (14–17). Despite controlling for several confounding factors in previous studies, it is important to acknowledge that residual confounding remains unavoidable. Consequently, there is a dearth of genetic evidence from extensive, population-based studies regarding the potential association between hypotension and an elevated risk of delirium.

Mendelian randomization (MR) employs genetic data to evaluate the unbiased associations between outcomes and exposures, benefiting from random classification of genetic variation during meiosis to mitigate confounding factors. If the genetic prediction level of exposures is correlated with disease outcomes, it indicates that there may be a potential causal relationship between exposures and outcomes (18). In this study, two-sample MR analyses were conducted using summary data from large-scale genome-wide association studies (GWAS) to investigate the causal influence between hypotension and delirium.

## 2 Materials and methods

### 2.1 Study design and data sources

Two-sample Mendelian randomization (MR) analyses were employed to investigate the potential causal relationship between outcome and exposure variables. Unlike the one-sample MR approach, the two-sample MR method incorporates data from separate and unrelated GWAS, thereby enhancing its efficacy and robustness. In the present investigation, exposure data was represented by hypotension, while delirium was considered as the outcome variable. Instrumental variables (IVs) in the shape of single-nucleotide polymorphisms (SNPs) were selected for subsequent analyses. The present study adhered to the following three crucial assumptions of the MR design: (1) All chosen IVs exhibited a high correlation with the exposure variable; (2) It is imperative that all selected IVs are devoid of any potential confounding factors that could impact the relationship between exposure and outcome; (3) Moreover, the chosen IVs should exclusively impact outcomes through the exposure variable and not through alternative pathways.

In this investigation, GWAS summarized data pertaining to hypotension and delirium were obtained from the FinnGen Consortium data repository.^1^ The hypotension dataset included 305,353 controls and 3,061 cases, hypotension is currently defined as when systolic pressure is consistently less than 90 mm Hg or when diastolic pressure is consistently 60 mm Hg or less and the unadjusted period prevalence of whole population was 0.85%. While the delirium (An etiologically nonspecific organic cerebral syndrome characterized by concurrent disturbances of consciousness and attention, perception, thinking, memory, psychomotor behavior, emotion, and the sleep–wake schedule. The duration is variable and the degree of severity ranges from mild to very severe.) dataset, which excluded cases induced by alcohol or other psychoactive substances, comprised 294,500 controls and 2,090 cases, and the unadjusted period prevalence of whole population was 0.76%. All study participants were of European ancestry, adjustments were made for sex, age, first 10 principal components and genotyping batch in the construction of the hypotension and delirium datasets, and explicit informed consent was secured from each individual.

### 2.2 IVs selection

The methodology utilized in this research involved the utilization of rigorously censored instrumental variables to examine the correlation between outcome and exposure. Given the restricted number of SNPs meeting the criteria for genome-wide significance in the incompletely summarized GWAS data, we adjusted the stringent association threshold to *p* < 5 × 10^−6^ (*F* > 10). SNPs demonstrating a significant association with the exposure were identified. Nevertheless, it is imperative to acknowledge the potential bias introduced by substantial linkage disequilibrium among these chosen SNPs. In order to mitigate this concern, a clumping process was employed with specific parameters (r^2^ < 0.01, clumping distance = 10,000 kb) to eliminate linkage disequilibrium among the included instrumental variables. Additionally, palindromic SNPs exhibiting intermediate allele frequencies were removed to ensure that the influence of SNPs on exposure aligned with the same allele as the effect on the outcome. Moreover, the PhenoScanner database was utilized to assess potential relationships between the selected SNPs and other traits, with significance thresholds established at the genome-wide level.

### 2.3 Statistical analysis

The present study utilized five different methodologies, including IVW, MR-Egger regression, simple mode, weighted median, and weight mode methods, to examine the genetic correlation between hypotension and delirium. The IVW method was chosen as the primary analysis due to its assumption of the validity of all SNPs utilized, leading to a more precise estimation. The Cochrane’s Q test was employed to evaluate heterogeneity in the association. Subsequently, the findings were visually depicted using scatter plots, forest plots, and funnel plots. In the scatter plot, the IVW method remained the primary focus, with a very small intercept indicating that confounding factors had minimal influence and did not affect the reliability of the results. A positive slope of the line indicated a risk factor, while a negative slope indicated a protective factor. Funnel plot was constructed to assess randomness, and if the IVs were symmetrically distributed along both sides of the IVW line, it indicated that MR conformed to Mendel’s second law of random grouping. The study utilized the MR-PRESSO global examination and MR-Egger intercept test to evaluate the presence of pleiotropy. MR-PRESSO was also employed to detect and remove outliers in the association, yielding an estimate. Additionally, a leave-one-out test was performed to assess the robustness of the results by examining the influence of individual SNPs on the association. All statistical analyses were carried out using R software (version 4.3.2) and the TwoSampleMR package, with a significance level set at *p* < 0.05.

## 3 Results

### 3.1 Instrumental variable selection

After initially setting the threshold at *p* < 5 × 10^−8^ and finding no SNPs meeting this criterion, the threshold was adjusted to 5 × 10^−6^, leading to the identification of 110 SNPs. Subsequently, SNPs with r^2^ > 0.001 were eliminated within a clumping distance of 10,000 kb, resulting in 8 SNPs. Further analysis revealed that rs111920693 exhibited significant deviation from the other SNPs in the leave-one-out examination, prompting its removal. Following a meticulous screening process, 7 SNPs were identified as IVs for our research. These SNPs were chosen according to their significant correlation with exposure data (*p* < 5 × 10^−6^, *F*-value >10) and their lack of correlation with each other (r^2^ < 0.01, clumping distance = 10,000 kb). The lowest F-value among these IVs was 1408.79. Detailed information on these IVs can be found in Table 1. Additionally, precautions were taken to exclude SNPs linked to outcomes and confounders (*p* < 1 × 10^−5^) using the Phenoscanner database, although no SNPs were ultimately removed during this process. The distribution of selected SNPs in clinical dataset can be found in Supplementary Table S1.

**Table 1:** Detailed statistics of selected SNPs for hypotension (exposure) and delirium (outcome).

rsids	beta	eaf	SE	*P*-value	R^2^	F statistic
rs144455898	0.5247	0.0068	0.1128	3.32E−06	0.0037	1408.79
rs62397195	0.2135	0.0547	0.0448	1.88E−06	0.0047	1782.984
rs62416431	0.1912	0.0679	0.0407	2.67E−06	0.0046	1750.467
rs141890899	−0.2423	0.0503	0.0527	4.26E−06	0.0056	2122.147
rs7170637	0.1274	0.2214	0.0267	1.87E−06	0.0056	2117.99
rs7218319	−0.1963	0.0842	0.0409	1.64E−06	0.0059	2248.016
rs2868934	0.1427	0.8401	0.0306	3.09E-06	0.0055	2071.487

SNP, single nucleotide polymorphism; SE, standard error; eaf, effect allele frequency.

### 3.2 Results of Mendelian randomization analysis

**Figure 1:**
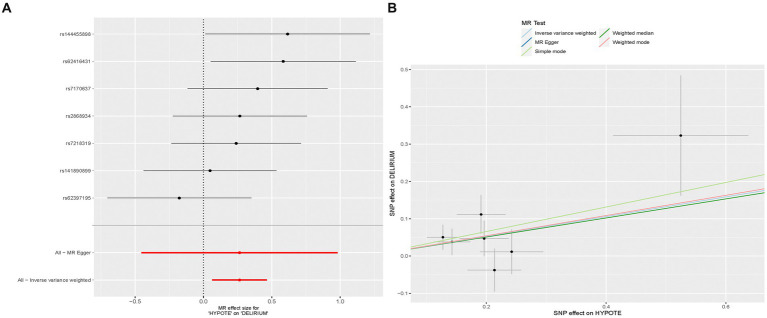
The association between hypotension and delirium. **(A)** Forest plot of causal effects of hypotension on delirium. **(B)** Scatter plot of causal effects of hypotension on delirium. The slope of the line represents the causal effect of each method.

In our study, we utilized the random-effects IVW method to explore the genetic correlation between hypotension and delirium. The findings revealed that hypotension was positively associated with delirium (*p* = 0.010, odds ratio [OR] [95% confidence interval (CI)] = 1.302 [1.066–1.592]) (Figures 1A,B). Although the *p*-values from the additional four methods did not reach statistical significance, their OR values were consistently above 1. The scatter plot of the present study (Figure 1B) depicted a positive linear trend for hypotension, indicating that heightened hypotension expression corresponded to an increased likelihood of delirium development. Forest plot (Figure 1A) was utilized to evaluate the predictive efficacy of each SNP locus in relation to exposure factors and outcomes. The solid dots on the left side of the plots denoted lower risk, whereas those on the right side denoted higher risk. Consistently, the forest plot finding showed solid dots predominantly on the right side, suggesting that increased hypotension was associated with a higher risk of delirium as determined by the IVW approach. Comprehensive results of our MR analyses, incorporating the five methods utilized, are detailed in Table 2.

**Table 2:** The MR results by five methods.

Exposure	Outcome	Method	SNP (*n*)	OR	OR95%CI		*P*-value
Hypotension	Delirium	MR Egger	7	1.302	0.635	2.671	0.503
Hypotension	Delirium	Weighted median	7	1.291	0.986	1.690	0.063
Hypotension	Delirium	IVW	7	1.302	1.066	1.592	0.010
Hypotension	Delirium	Simple mode	7	1.389	0.895	2.157	0.193
Hypotension	Delirium	Weighted mode	7	1.312	0.885	1.944	0.225

MR, Mendelian randomization; SNP, single nucleotide polymorphism; IVW, Inverse variance weighted; OR, odds ratio; CI, confidence interval.

**Figure 2:**
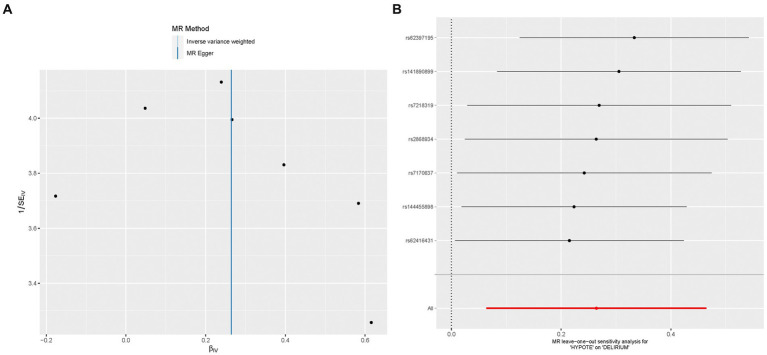
The effect size for hypotension on delirium. **(A)** The funnel plot showed that the SNPs were symmetric, indicating that there was no heterogeneity in the association. **(B)** The leave-one-out test showed that the result was not affected by single influential SNP, so this association was stable.

The study utilized the Cochrane’s Q test to assess the presence of heterogeneity in the relationship between hypotension and delirium, with results indicating no significant evidence supporting such heterogeneity (*p* = 0.379). Concurrently, The assessment of instrumental variable randomness was conducted and visually depicted using a funnel plot (Figure 2A) which demonstrated a balanced distribution of IVs on either side of the IVW line, validating the adherence of the MR analysis to the principles of MR grouping. Both the MR-intercept and MR-PRESSO global tests yielded a high *p*-value of 0.999, suggesting the absence of horizontal pleiotropy in the association. Moreover, the MR-PRESSO analysis did not identify any outliers in the MR analysis. The main goal of leave-one-out examination is to determine if the line connecting the black dots lies entirely on one side of the dotted line without any noticeable bias points. The result of the leave-one-out analysis (Figure 2B) in the present study indicated the absence of significant bias points in the data, consequently, these findings can be deemed stable and robust.

## 4 Discussion

In the present study, we employed two-sample MR analyses utilizing data obtained from a large-scale GWAS to explore the potential causal connections between hypotension and delirium. Our findings suggest that individuals with a genetic predisposition for hypotension may have a higher risk of developing delirium. The correlation between hypotension and delirium indicates that interventions aimed at improving hypotension before surgery could potentially reduce the incidence and severity of delirium in hospitalized patients. This could ultimately lead to a decrease in long-term cognitive decline and potential adverse clinical results.

There is an increasing amount of evidence suggesting a correlation between intraoperative hypotension and an elevated risk of postoperative delirium. Duan et al. (15) conducted a population-based cohort study involving 605 elderly patients undergoing thoracic and orthopedic surgery, and their findings revealed that intraoperative hypotension lasting longer than 5 min significantly raised the likelihood of postoperative delirium. Similarly, in a retrospective study conducted by Wachtendorf et al. (16), involving a substantial sample size of 316,717 surgical patients, it was determined that individuals undergoing noncardiac surgery exhibited a duration-dependent escalation in the likelihood of experiencing postoperative delirium when their mean arterial pressure (MAP) fell below 55 mm Hg. Ushio et al. (19) conducted a study wherein they provided evidence that an extended cumulative duration of intraoperative hypotension, characterized by a MAP below 75 mmHg, significantly heightened the likelihood of postoperative delirium in adult patients following cardiopulmonary bypass. Similarly, Maheshwari et al. (20) discovered that an intraoperative time-weighted average of MAP below 65 mmHg amplified the risks of postoperative delirium among patients who were transferred to the intensive care unit (ICU) subsequent to surgery. These results suggest that promptly addressing intraoperative hypotension could potentially reduce the risk of postoperative delirium.

Nevertheless, the establishment of a causal association between hypotension and delirium remains inconclusive based on observational studies. The exploration of this relationship through prospective randomized controlled trials is unfeasible due to ethical constraints, thereby restricting the evaluation to retrospective observation. However, it is important to acknowledge that retrospective research possesses inherent limitations, including a low level of evidence and the potential for data selection bias. GWASs have emerged as a valuable methodology for investigating complex diseases, surpassing the limitations of single-gene association studies by identifying individual or groups of genes. By confirming prior research and introducing novel perspectives, GWASs have proven instrumental in advancing our understanding of intricate disorders. In light of a comprehensive GWAS conducted, our study provides strong genetic proof that supports a causal relationship between hypotension and delirium, two complex and interconnected conditions.

A decline in MAP results in a reduction in cerebral perfusion pressure and a decrease in mean cerebral blood flow velocity, potentially rendering neuronal cells more susceptible to ischemic damage. A research investigation focusing on hypotension throughout the initiation of general anesthesia among patients underwent neurosurgical operations demonstrated that the velocity of the middle cerebral artery decreased in response to a decline in MAP throughout anesthesia induction, but subsequently increased upon the administration of norepinephrine via bolus infusion (21). Research suggests that reduced baseline cerebral blood flow, often seen in neurosurgical patients, as well as decreased cerebral blood flow velocity and brain tissue oxygenation among patients suffered septic shock and those undergoing cardiac surgery, are identified as independent hazard variables for the development of delirium (22). Consequently, the occurrence of postoperative delirium caused by hypotension can be attributed to neuronal injuries, which are regarded as potential mechanisms.

This study exhibits numerous significant strengths. Primarily, to the best of our knowledge, our research represents the first investigation into the causal association between hypotension and delirium, utilizing a comprehensive large-scale GWAS. The utilization of the two-sample MR method allows us to overcome limitations present in some observational studies, such as confounding variables and various biases. Secondly, all IVs utilized in the MR analysis were subjected to thorough screening, with the minimum *F*-value achieving a noteworthy 1408.79, thus guaranteeing the highest level of precision and accuracy in the resultant findings. Subsequently, a variety of techniques were utilized to evaluate the sensitivity, horizontal pleiotropy, and heterogeneity. These analyses collectively indicated that the relationship between hypotension and delirium remained consistent and strong.

However, it is crucial to recognize the constraints of this study. Firstly, the restriction to individuals of European descent in the GWAS raises concerns about the applicability of our results to varied populations and geographic areas, warranting additional research. Secondly, due to the reliance on data from the underlying GWAS meta-analyses, the present MR analysis was unable to perform stratified analyses based on factors such as ethnicities, countries, or age groups. Thirdly, the known causes of hypotension during surgery are largely not genetically determined (bleeding, vasodilatation, cardiac dysfunction), therefore, additional research is warranted to investigate the potential correlation between genetic predisposition to hypotension and the occurrence of delirium in patients, taking into consideration potential confounding variables such as bleeding, vasodilation, and cardiovascular dysfunction. Consequently, the findings of hypotension effects in the present MR study cannot be generalizable to populations with varying characteristics, such as age and ethnicity.

## 5 Conclusion

The present study has identified a causal relationship between hypotension and delirium, potentially supported by shared neural pathways. These findings contribute to a deeper understanding of the genetic and biological mechanisms involved in the development of hypotension and delirium.

## Data availability statement

The original contributions presented in the study are included in the article/Supplementary material, further inquiries can be directed to the corresponding authors.

## Author contributions

CW: Writing – review & editing, Writing – original draft, Methodology, Conceptualization. JW: Writing – review & editing, Writing – original draft, Software, Methodology, Formal analysis, Data curation. YL: Writing – review & editing, Writing – original draft, Software, Formal analysis. ZL: Writing – review & editing, Writing – original draft, Software. NL: Writing – review & editing, Writing – original draft, Visualization, Supervision, Conceptualization. MC: Writing – review & editing, Writing – original draft, Visualization, Supervision, Funding acquisition, Conceptualization.

## Footnotes

^1^ https://r9.finngen.fi/
