# Supplementary material for: The genetic relationship between hypotension and delirium: a Mendelian randomization study
Source: Front Neurol. 2024 Jul 17;15:1408956. doi: 10.3389/fneur.2024.1408956 (PMC11288944; doi:10.3389/fneur.2024.1408956)
Supplement: Supplementary file 1 [file Table_1.docx]

**Supplementary Table S1. The distribution of selected SNPs in clinical dataset**

| snp | hg19_coordinates | hg38_coordinates | a1 | a2 | trait | efo | study | pmid | ancestry | year | beta | se | p | n | n_cases | n_controls | dataset |
| --- | --- | --- | --- | --- | --- | --- | --- | --- | --- | --- | --- | --- | --- | --- | --- | --- | --- |
| rs7170637 | chr15:22969232 | chr15:22903836 | A | G | Platelet distribution width | EFO_0004586 | Astle W | 27863252 | European | 2016 | 0.02609 | 0.00488 | 9.02E-08 | 173480 | 0 | 173480 | Astle-W_Blood-Cell-Traits_EUR_2016 |
| rs7218319 | chr17:44126365 | chr17:46048999 | C | T | Eosinophil count | EFO_0004586 | Astle W | 27863252 | European | 2016 | 0.03428 | 0.004197 | 3.16E-16 | 173480 | 0 | 173480 | Astle-W_Blood-Cell-Traits_EUR_2016 |
| rs7218319 | chr17:44126365 | chr17:46048999 | C | T | Eosinophil percentage of granulocytes | EFO_0004586 | Astle W | 27863252 | European | 2016 | 0.04216 | 0.00421 | 1.35E-23 | 173480 | 0 | 173480 | Astle-W_Blood-Cell-Traits_EUR_2016 |
| rs7218319 | chr17:44126365 | chr17:46048999 | C | T | Eosinophil percentage of white cells | EFO_0004586 | Astle W | 27863252 | European | 2016 | 0.0399 | 0.004192 | 1.74E-21 | 173480 | 0 | 173480 | Astle-W_Blood-Cell-Traits_EUR_2016 |
| rs7218319 | chr17:44126365 | chr17:46048999 | C | T | Granulocyte count | EFO_0004586 | Astle W | 27863252 | European | 2016 | -0.02073 | 0.004223 | 9.19E-07 | 173480 | 0 | 173480 | Astle-W_Blood-Cell-Traits_EUR_2016 |
| rs7218319 | chr17:44126365 | chr17:46048999 | C | T | Hematocrit | EFO_0004586 | Astle W | 27863252 | European | 2016 | -0.0439 | 0.004155 | 4.29E-26 | 173480 | 0 | 173480 | Astle-W_Blood-Cell-Traits_EUR_2016 |
| rs7218319 | chr17:44126365 | chr17:46048999 | C | T | Hemoglobin concentration | EFO_0004586 | Astle W | 27863252 | European | 2016 | -0.04196 | 0.004173 | 8.80E-24 | 173480 | 0 | 173480 | Astle-W_Blood-Cell-Traits_EUR_2016 |
| rs7218319 | chr17:44126365 | chr17:46048999 | C | T | High light scatter percentage of red cells | EFO_0004586 | Astle W | 27863252 | European | 2016 | -0.02528 | 0.004217 | 2.04E-09 | 173480 | 0 | 173480 | Astle-W_Blood-Cell-Traits_EUR_2016 |
| rs7218319 | chr17:44126365 | chr17:46048999 | C | T | High light scatter reticulocyte count | EFO_0004586 | Astle W | 27863252 | European | 2016 | -0.03271 | 0.004217 | 8.82E-15 | 173480 | 0 | 173480 | Astle-W_Blood-Cell-Traits_EUR_2016 |
| rs7218319 | chr17:44126365 | chr17:46048999 | C | T | Lymphocyte percentage of white cells | EFO_0004586 | Astle W | 27863252 | European | 2016 | 0.02819 | 0.004191 | 1.75E-11 | 173480 | 0 | 173480 | Astle-W_Blood-Cell-Traits_EUR_2016 |
| rs7218319 | chr17:44126365 | chr17:46048999 | C | T | Mean corpuscular hemoglobin | EFO_0004586 | Astle W | 27863252 | European | 2016 | 0.02043 | 0.004166 | 9.43E-07 | 173480 | 0 | 173480 | Astle-W_Blood-Cell-Traits_EUR_2016 |
| rs7218319 | chr17:44126365 | chr17:46048999 | C | T | Mean corpuscular volume | EFO_0004586 | Astle W | 27863252 | European | 2016 | 0.02142 | 0.004154 | 2.53E-07 | 173480 | 0 | 173480 | Astle-W_Blood-Cell-Traits_EUR_2016 |
| rs7218319 | chr17:44126365 | chr17:46048999 | C | T | Mean platelet volume | EFO_0004586 | Astle W | 27863252 | European | 2016 | 0.0252 | 0.00425 | 3.02E-09 | 173480 | 0 | 173480 | Astle-W_Blood-Cell-Traits_EUR_2016 |
| rs7218319 | chr17:44126365 | chr17:46048999 | C | T | Monocyte count | EFO_0004586 | Astle W | 27863252 | European | 2016 | -0.01969 | 0.004203 | 2.81E-06 | 173480 | 0 | 173480 | Astle-W_Blood-Cell-Traits_EUR_2016 |
| rs7218319 | chr17:44126365 | chr17:46048999 | C | T | Myeloid white cell count | EFO_0004586 | Astle W | 27863252 | European | 2016 | -0.02116 | 0.004234 | 5.85E-07 | 173480 | 0 | 173480 | Astle-W_Blood-Cell-Traits_EUR_2016 |
| rs7218319 | chr17:44126365 | chr17:46048999 | C | T | Neutrophil count | EFO_0004586 | Astle W | 27863252 | European | 2016 | -0.02439 | 0.004213 | 7.04E-09 | 173480 | 0 | 173480 | Astle-W_Blood-Cell-Traits_EUR_2016 |
| rs7218319 | chr17:44126365 | chr17:46048999 | C | T | Neutrophil percentage of granulocytes | EFO_0004586 | Astle W | 27863252 | European | 2016 | -0.04149 | 0.004211 | 6.63E-23 | 173480 | 0 | 173480 | Astle-W_Blood-Cell-Traits_EUR_2016 |
| rs7218319 | chr17:44126365 | chr17:46048999 | C | T | Neutrophil percentage of white cells | EFO_0004586 | Astle W | 27863252 | European | 2016 | -0.03121 | 0.004198 | 1.06E-13 | 173480 | 0 | 173480 | Astle-W_Blood-Cell-Traits_EUR_2016 |
| rs7218319 | chr17:44126365 | chr17:46048999 | C | T | Red blood cell count | EFO_0004586 | Astle W | 27863252 | European | 2016 | -0.05041 | 0.004186 | 2.10E-33 | 173480 | 0 | 173480 | Astle-W_Blood-Cell-Traits_EUR_2016 |
| rs7218319 | chr17:44126365 | chr17:46048999 | C | T | Red cell distribution width | EFO_0004586 | Astle W | 27863252 | European | 2016 | -0.04641 | 0.004164 | 7.59E-29 | 173480 | 0 | 173480 | Astle-W_Blood-Cell-Traits_EUR_2016 |
| rs7218319 | chr17:44126365 | chr17:46048999 | C | T | Reticulocyte count | EFO_0004586 | Astle W | 27863252 | European | 2016 | -0.03412 | 0.004223 | 6.57E-16 | 173480 | 0 | 173480 | Astle-W_Blood-Cell-Traits_EUR_2016 |
| rs7218319 | chr17:44126365 | chr17:46048999 | C | T | Reticulocyte fraction of red cells | EFO_0004586 | Astle W | 27863252 | European | 2016 | -0.02336 | 0.004221 | 3.11E-08 | 173480 | 0 | 173480 | Astle-W_Blood-Cell-Traits_EUR_2016 |
| rs7218319 | chr17:44126365 | chr17:46048999 | C | T | Sum basophil neutrophil counts | EFO_0004586 | Astle W | 27863252 | European | 2016 | -0.02455 | 0.00422 | 5.93E-09 | 173480 | 0 | 173480 | Astle-W_Blood-Cell-Traits_EUR_2016 |
| rs7218319 | chr17:44126365 | chr17:46048999 | C | T | Sum eosinophil basophil counts | EFO_0004586 | Astle W | 27863252 | European | 2016 | 0.0318 | 0.004202 | 3.80E-14 | 173480 | 0 | 173480 | Astle-W_Blood-Cell-Traits_EUR_2016 |
| rs7218319 | chr17:44126365 | chr17:46048999 | C | T | Sum neutrophil eosinophil counts | EFO_0004586 | Astle W | 27863252 | European | 2016 | -0.02054 | 0.004217 | 1.11E-06 | 173480 | 0 | 173480 | Astle-W_Blood-Cell-Traits_EUR_2016 |
| rs7218319 | chr17:44126365 | chr17:46048999 | C | T | Lumbar spine bone mineral density in females | EFO_0008474 | GEFOS | 22504420 | Mixed | 2012 | NA | NA | 3.95E-06 | 21720 | 0 | 21720 | GEFOS_LSBMD-F_Mixed_2012 |
| rs7218319 | chr17:44126365 | chr17:46048999 | C | T | Lumbar spine bone mineral density | EFO_0007701 | GEFOS | 22504420 | Mixed | 2012 | NA | NA | 1.56E-07 | 31800 | 0 | 31800 | GEFOS_LSBMD_Mixed_2012 |
